# P179: Establishing a collaboration with industry to support HAI reduction

**DOI:** 10.1186/2047-2994-2-S1-P179

**Published:** 2013-06-20

**Authors:** D Pittet, C Kilpatrick, E Kelley, B Allegranzi, S Bagheri-Nejad

**Affiliations:** 1 University Hospitals Geneva, Geneva, Switzerland; 2 World Health Organisation, 1211 Geneva, Switzerland

## Introduction

Collaboration with industry focused on health improvement is often viewed sceptically while existing examples show a clear public health benefit. An approach was adopted to explore the potential for establishing a collaborative with the World Health Organisation (WHO) Patient Safety Programme.

## Objectives

To establish a transparent WHO industry collaborative for the benefit of patients, avoiding a focus on the potential for commercial gain.

## Methods

Between 2007 and 2012 a number of steps were undertaken; 1) scoping of the potential for a collaborative, the benefits to patients and to establish an aim, which included desk research and multidisciplinary/agency discussions 2) interviews with WHO legal department 3) an announcement to potential participants and informal collaborative face-to-face interviews with interested parties to strengthen a realistic patient-focused aim.

## Results

A formal proposal was approved by WHO legal department in 2012, which includes the overall aim of improving systems, education and research in order to provide a public health benefit by reducing health care-associated infections (HAI). Criteria for participation are clear and a code of conduct and finance details are outlined. Implementation was approved as a protected web-based platform to allow for targeted interaction, with a first year aim of evaluating this. A total of 14 companies from industries related to improving patient safety through hand hygiene responded to the call from WHO and have signed up to the code of conduct and provided finance. An editorial and note for the media announcing the collaborative and its name ‘Private Organisations for Patient Safety (POPS)’ was issued in 2012 to support transparency. One formal collaborative project has been undertaken.

## Conclusion

A transparent collaborative has been established and funded which presents a clear public health benefit and ensures that those involved are focused on corporate social responsibility. As the plan for the first year is to evaluate the platform method of working, this will inform next steps as to whether it is possible to undertake fair and equitable project working which can significantly contribute to reducing HAI in the long term.

## Disclosure of interest

None declared

